# Swimming in Allergens?: Pool Use and Asthma

**Published:** 2006-10

**Authors:** Angela Spivey

Atopic asthma (inflammation of the airways caused by exposure to airborne allergens) has become increasingly prevalent since the 1960s and is now the most common chronic childhood disease in the United States and many other industrialized countries. The cause for the rise is unclear, though many hypotheses have been put forth. Now researchers provide new findings that further support one proposed reason—increased use of indoor chlorinated swimming pools by children **[*EHP* 114:1567–1573; Bernard et al.]**.

The researchers studied 341 children aged 10–13 years who had attended, at varying rates, three indoor pools in Brussels, Belgium. Ambient levels of a highly reactive chlorine by-product, trichloramine, ranged from 0.25 to 0.54 mg/m^3^ at these pools. Trichloramine is created when chlorine reacts with organic matter such as sweat or urine. The researchers administered various tests to the participants, including a questionnaire about health history and pool attendance, an exercise-induced bronchoconstriction test, and a measurement of total serum and aeroallergen-specific IgE (a mediator of atopic asthma).

Forty children had asthma, as indicated by previous doctor diagnosis or the exercise-induced bronchoconstriction test. Cumulative time spent at swimming pools emerged as one of the most consistent predictors of asthma, just after family history of asthma or hay fever and atopy (a genetic tendency toward developing IgE-mediated allergies).

Time spent at pools was associated with increased incidence of asthma only in children with elevated serum IgE. All the effects were dose-related and most strongly linked to pool attendance before the age of about 7 years, suggesting that attendance at indoor chlorinated pools, especially by young children, interacts with atopic status to promote the development of childhood atopic asthma.

The scientists suggest a possible mechanism—that chlorine by-products such as trichloramine disrupt the protective epithelial barriers of the respiratory tract, allowing allergens to enter the lungs. This idea is supported by earlier findings from the same team that children who used an indoor pool showed increased levels of blood markers that indicated damage to these protective membranes, indeed after as little as 1 hour of exposure.

The findings suggest that regular attendance at pools, especially during early life, can promote the development of atopic asthma. Given that atopic asthma is the form of the disease that appears largely responsible for the childhood asthma epidemic and is a chronic disease that greatly affects quality of life, the study points to the need for preventive measures, the authors state. One option is that children younger than 7, especially those with allergies, could avoid strongly chlorinated swimming pools, as identified by a strong smell of chlorine at the surface of the water (for outdoor pools) or inside the enclosure (for indoor pools).

## Figures and Tables

**Figure:**
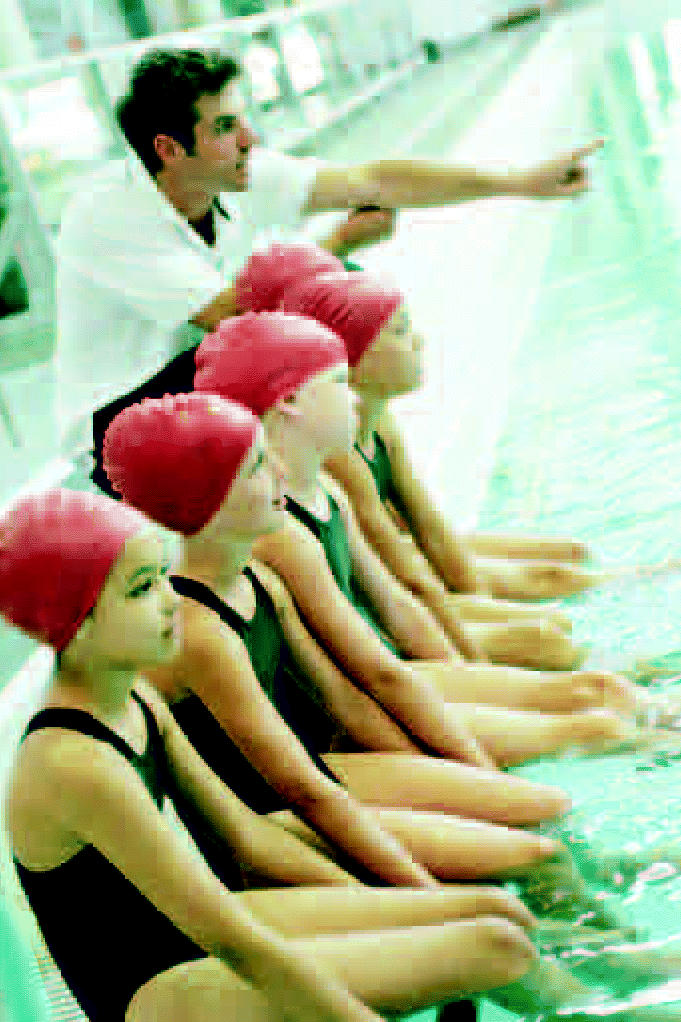
Pooling data Regular exposure to chlorine at indoor pools may contribute to atopic asthma.

